# Disseminated Tuberculosis With Presumptive Tricuspid Valve Endocarditis Presenting as Culture-Negative Infective Endocarditis: A Clinical Diagnostic Challenge

**DOI:** 10.7759/cureus.109757

**Published:** 2026-05-27

**Authors:** Shaurya Tewari, Shrikanth Jantli, Mahadev Meena, Sagar Khadanga

**Affiliations:** 1 Internal Medicine, All India Institute of Medical Sciences, Bhopal, Bhopal, IND

**Keywords:** bcne, cbnaat, disseminated tb, infective endocarditis, presumptive tuberculous endocarditis, puo

## Abstract

Tuberculous endocarditis is a rare manifestation of *Mycobacterium tuberculosis* and may present as blood culture-negative infective endocarditis (BCNE). A 34-year-old male with no prior documented immunosuppressive illness or comorbidities, resident of Raisen district, Madhya Pradesh - a high tuberculosis (TB)-burden region - presented with a three-month history of intermittent high-grade fever, night sweats, and significant weight loss. Examination revealed pallor and tender cervical lymphadenopathy. Laboratory evaluation showed anaemia, leukopenia (WBC 2.85 × 10³/μL), elevated ferritin (640.60 ng/mL), erythrocyte sedimentation rate (ESR), and C-reactive protein (CRP), while repeated pre-treatment blood and urine cultures were sterile. Chest radiograph revealed bilateral hilar lymphadenopathy. Echocardiography, performed in the context of a newly auscultated tricuspid regurgitation murmur and persistent fever despite broad-spectrum antibiotics, demonstrated flail tricuspid valve leaflets with vegetations consistent with infective endocarditis. Bone marrow biopsy showed epithelioid granulomas with Langhans-type giant cells. Contrast-enhanced computed tomography (CECT) of the thorax and abdomen revealed necrotic mediastinal lymphadenopathy, hepatic granulomas, abdominal (periportal and paraaortic) lymphadenopathy, and pulmonary tree-in-bud nodules. The spleen was unremarkable. Bronchoalveolar lavage cartridge-based nucleic acid amplification test (CBNAAT) detected rifampicin-sensitive *M. tuberculosis*, confirming disseminated TB. A diagnosis of disseminated TB with presumptive tricuspid valve endocarditis was made. The patient responded rapidly to first-line antituberculous therapy (HRZE (isoniazid, rifampin, pyrazinamide, and ethambutol) × two months, followed by HR (isoniazid and rifampin) × 10 months; total 12 months) with adjunctive corticosteroids, becoming afebrile within a week. We report this case to highlight *M. tuberculosis* as a rare but important aetiology of culture-negative endocarditis, particularly in patients from TB-endemic regions, and to illustrate the clinical reasoning required when valve tissue cannot be obtained for confirmation.

## Introduction

The syndrome of pyrexia of unknown origin (PUO) remains a clinical challenge to date. Infectious causes account for 17-35% of PUO cases, with infective endocarditis (IE), particularly culture-negative endocarditis, representing one of the most important and diagnostically demanding etiologies. Other common causes of PUO in patients without documented immunosuppression include discitis, osteomyelitis, occult abscesses, and infected implanted devices. [1]

IE is typically diagnosed using the modified Duke Criteria [2], which rely heavily on positive blood cultures as a major criterion. Blood culture-negative infective endocarditis (BCNE) - defined as IE in which standard blood cultures remain sterile despite adequate pre-treatment sampling - occurs in approximately 11.65% of IE cases [3]. In BCNE, the absence of a microbiological major criterion significantly narrows the diagnostic pathway, and clinicians must systematically consider a distinct set of fastidious or intracellular pathogens, including Coxiella burnetii, Bartonella spp., Brucella, fungal organisms, and non-infective (marantic) vegetations, in addition to mycobacterial causes.

Cardiovascular involvement in tuberculosis (TB) occurs in 0.14-2% of TB patients [4]. Tuberculous pericarditis is the most common form of cardiac TB, while endocardial involvement is far rarer, mainly documented in autopsy series. When it does occur, tuberculous endocarditis arises through hematogenous seeding of the cardiac valves during episodes of mycobacteremia in the setting of miliary or disseminated disease [5]. Critically, Mycobacterium tuberculosis is rarely considered in the initial differential of BCNE even in TB-endemic regions, because standard blood cultures do not grow mycobacteria under conventional conditions and confirmatory valve tissue is seldom obtainable. Missing this diagnosis carries significant consequences - empirical antibacterial therapy directed at conventional IE pathogens will be ineffective, and the underlying disseminated TB will progress untreated. The changing profile of IE in India further compounds this diagnostic challenge for clinicians [6].

We report this case of disseminated TB presenting as BCNE to highlight M. tuberculosis as a rare but important etiology of culture-negative endocarditis, particularly in patients from TB-endemic regions, and to present a structured clinical and microbiological reasoning framework for approaching a cardiac vegetation in a patient with confirmed disseminated TB when valve tissue cannot be obtained for confirmation.

## Case presentation

A 34-year-old male with no known comorbidities, residing in Raisen district, Madhya Pradesh (a high TB-burden region per WHO and national TB programme data), with no recent travel history outside the region, presented with a three-month history of high-grade fever. The fever was intermittent, associated with chills, rigors, and drenching night sweats. He had lost over 8 kilograms over three months. No prior history of immunosuppressive illness, corticosteroid use, or known haematological disorder was elicited. No potentially diagnostic clues for localisation were found on history.

On examination, the patient appeared toxic and was febrile. Examination revealed pallor and multiple right-sided cervical lymph nodes, firm and tender, measuring approximately 0.5 to 1.5 cm in greatest dimension. Cardiovascular examination at this stage was unremarkable. Respiratory, neurological, and abdominal examinations were also unremarkable.

Obligatory investigations for PUO evaluation were performed, including complete blood count (CBC) with peripheral smear; erythrocyte sedimentation rate (ESR) and C-reactive protein (CRP); liver function tests (LFT) and renal function tests (RFT); blood cultures (three sets, all drawn prior to antibiotic initiation) and urine routine with culture; chest X-ray; and abdominal ultrasonography. CBC was suggestive of normocytic normochromic anaemia with leukopenia (WBC 2.85 × 10³/μL) and relative lymphopenia (lymphocytes 17.9%). Kidney function tests were normal. Liver function tests showed mildly elevated alkaline phosphatase (ALP 190.4 U/L) and gamma-glutamyl transferase (GGT 114.6 U/L) with hypoalbuminemia (albumin 2.89 g/dL) and elevated globulins (4.09 g/dL), a pattern consistent with systemic granulomatous disease. Ferritin was notably elevated at 640.60 ng/mL (reference 22-275 ng/mL; approximately 2.3 times the upper limit of normal), consistent with active macrophage activation in granulomatous disease. Hyperferritinemia in this clinical context prompted consideration of haemophagocytic lymphohistiocytosis (HLH); however, the ferritin level was substantially below the extreme hyperferritinemia typically seen in HLH (often >10,000 ng/mL), and bone marrow biopsy subsequently showed no haemophagocytosis, making HLH unlikely. All three pre-treatment blood culture sets and urine cultures were sterile. Human immunodeficiency virus (HIV), hepatitis B, hepatitis C serology, antinuclear antibodies (ANA), rheumatoid factor (RA), and Brucella IgG were all negative. Inflammatory markers were elevated (Table 1).

**Table 1:** List of investigations performed * Ferritin 640.60 ng/mL (∼2.3× upper limit of normal): Hyperferritinemia is a recognized feature of active granulomatous disease. HLH was considered but excluded (no hemophagocytosis on bone marrow biopsy; ferritin substantially below levels typical for HLH). † Elevated ALP and GGT with hypoalbuminemia suggest hepatic granulomatous involvement. ‡ Hypoalbuminemia consistent with a systemic granulomatous inflammatory state. ESR – Erythrocyte sedimentation rate; AST – Aspartate aminotransferase; ALT – Alanine aminotransferase; ALP – Alkaline phosphatase; GGT – Gamma-glutamyl transferase; BAL – Bronchoalveolar lavage; CBNAAT – Cartridge-based nucleic acid amplification test; HIV – Human immunodeficiency virus; ANA – Antinuclear antibody; FNAC – Fine needle aspiration cytology; CECT – Contrast-enhanced computed tomography; LVEF – Left ventricular ejection fraction; HLH – Hemophagocytic lymphohistiocytosis.

Investigation	Result	Unit	Reference Range
ESR	63	mm/Hour	0-10
White Blood Cell Count	2.85	Thousand/μL	4-11
Neutrophils	73.3	%	40-70
Lymphocytes	17.9	%	20-40
Monocytes	7.4	%	2-8
Eosinophils	0.7	%	1-6
Basophils	0.7	%	0-1
Haemoglobin	8.9	gm/dL	11-15
Mean Cell Volume	84.4	fL	76-93
Platelet Count	224	Thousand/μL	150-450
C-Reactive Protein	43.41	mg/L	<5.00
Ferritin *	640.60	ng/mL	22-275
Urea	12.13	mg/dL	20.0-40.0
Creatinine	0.71	mg/dL	0.6-1.2
Sodium	132.02	mmol/L	136-145
Potassium	3.78	mmol/L	3.5-5.1
Chloride	99.12	mmol/L	98-107
Total Bilirubin	0.38	mg/dL	0.3-1.2
Direct Bilirubin	0.09	mg/dL	<0.2
Indirect Bilirubin	0.29	mg/dL	0.20-1.0
AST	38.5	U/L	<50
ALT	39.9	U/L	<50
ALP †	190.4	U/L	30-120
Total Protein	6.98	g/dL	6.6-8.3
Albumin ‡	2.89	g/dL	3.5-5.2
Globulin	4.09	g/dL	1.9-3.7
A/G Ratio	0.71	-	1-1.7
GGT †	114.6	U/L	<55
Peripheral Blood Smear	Predominantly normocytic normochromic RBCs	-	-
BAL CBNAAT	Positive	-	-
RIF Resistance Result	Not Detected	-	-
First-Line Probe Assay	Rifampicin (R): Sensitive; Isoniazid (KatG): Sensitive; Isoniazid (InhA): Sensitive	-	-
Blood Culture (3 pre-treatment sets)	No Growth in Culture	-	-
Hepatitis B and C	Non-Reactive	-	-
HIV	Non-Reactive	-	-
ANA	Negative	-	-
RA Factor	<9.62	-	<10
Brucella IgG	Negative	-	-
Bone Marrow Biopsy	Normocellular for age. Ill-formed epithelioid cell granulomas with Langhans giant cells and lymphocyte cuffing	-	-
FNAC of Lymph Node	Polymorphous population of lymphoid cells. No granulomas, no atypia or malignant cells (reactive lymphadenitis)	-	-
CECT Abdomen & Thorax	Multiple conglomerate necrotic lymph nodes with calcifications, hepatic granulomas, periportal and paraaortic lymphadenopathy, tree-in-bud opacities in the left lower lobe	-	-
2D Echocardiogram	Flail tricuspid valve with vegetation (~1.2 × 0.8 cm), moderate tricuspid regurgitation, LVEF 65%	-	-

**Figure 1:**
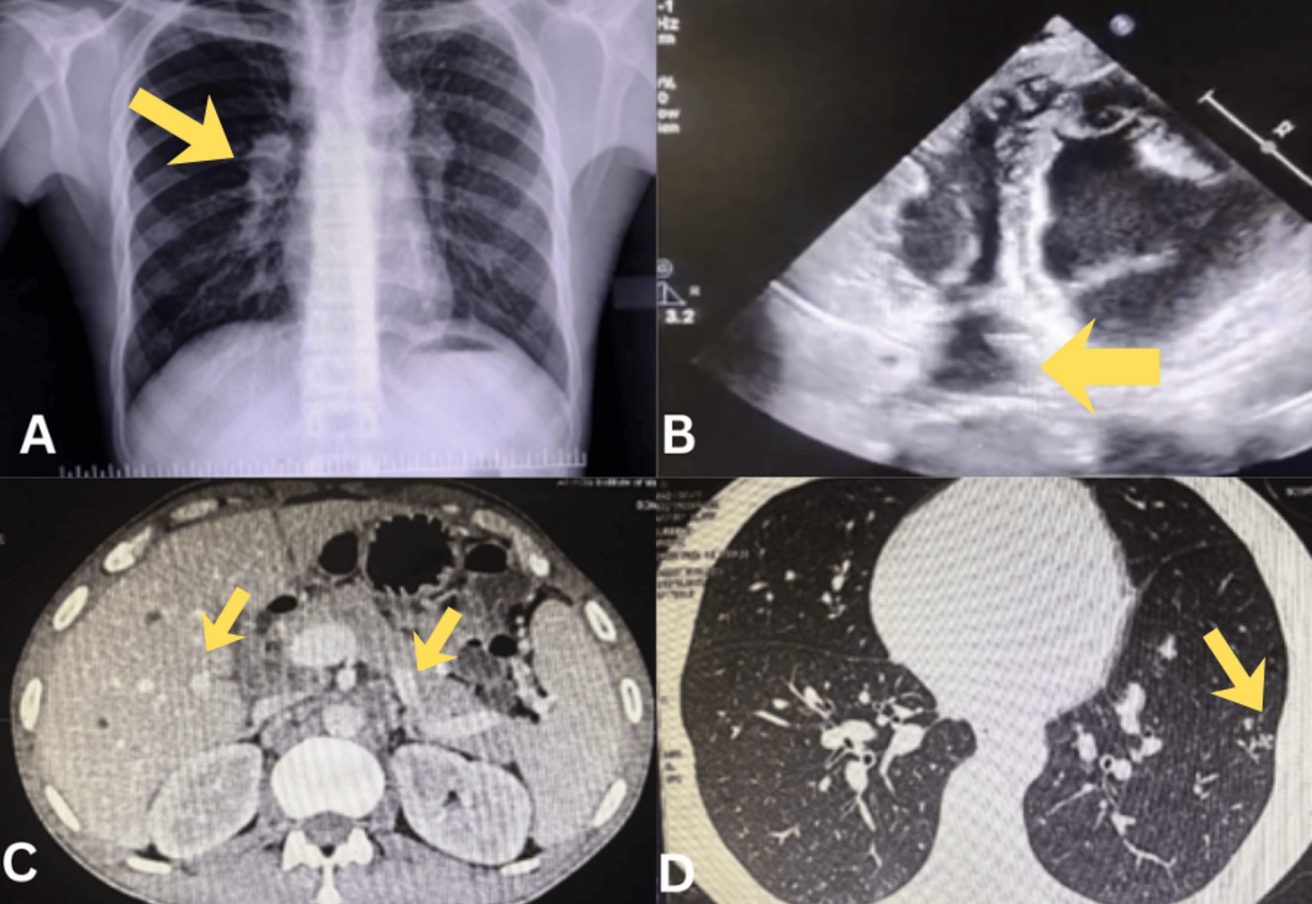
(A) Chest X-ray showing bilateral hilar lymphadenopathy (arrows indicate enlarged bilateral hilar nodes, right greater than left). (B) Transthoracic 2D echocardiogram showing a mobile vegetation (arrow) attached to the tricuspid valve leaflets, consistent with infective endocarditis; vegetation measured approximately 1.2 × 0.8 cm. (C) Contrast-enhanced computed tomography (CECT) abdomen showing conglomerate heterogeneously enhancing necrotic lymph nodes in the periportal, pre-aortic, and paraaortic locations (arrows), with multiple sub-centimetric non-enhancing hypodense lesions in the liver parenchyma consistent with hepatic granulomas. (D) CECT thorax showing centrilobular nodules with a tree-in-bud appearance in the left lower lobe (arrows), consistent with endobronchial spread of tuberculosis. Mediastinal lymphadenopathy is also visible at the carinal level.

Chest X-ray showed bilateral hilar lymphadenopathy (Figure 1A). Fine needle aspiration cytology (FNAC) of the right cervical lymph node showed a polymorphous population of lymphoid cells with no granulomas, no atypia, and no malignant cells, consistent with reactive lymphadenitis. While this result did not support a specific diagnosis, it is important to note that FNAC has a reported sensitivity of only 40-60% for granulomatous lymphadenitis due to sampling limitations and the patchy distribution of granulomas [7]; therefore, a non-diagnostic FNAC does not exclude TB in a high-prevalence setting. In retrospect, excisional biopsy, which carries substantially higher sensitivity, would have been the preferred next step.

In the context of a newly auscultated soft tricuspid regurgitation murmur and ongoing fever despite empirical broad-spectrum intravenous antibiotics (ceftriaxone, vancomycin, and gentamicin), a transthoracic echocardiogram was performed. This revealed a flail tricuspid valve with a mobile vegetation measuring approximately 1.2 × 0.8 cm attached to the tricuspid leaflets, with moderate tricuspid regurgitation and a preserved left ventricular ejection fraction (LVEF) of 65%. These findings were consistent with IE (Figure 1B). Intravenous antibiotics were continued empirically. Serial blood cultures obtained after 72 hours of antibiotic initiation were also sterile; these post-antibiotic cultures are of limited diagnostic value and are included only for completeness.

Given ongoing diagnostic uncertainty and the sterile culture results, bone marrow aspiration and biopsy were performed as a less-invasive approach to assess for disseminated disease (excisional lymph node biopsy was deferred in view of the patient’s clinical frailty at presentation). The bone marrow biopsy showed a normocellular marrow for age, with ill-formed epithelioid cell granulomas with Langhans-type giant cells and lymphocyte cuffing, highly suggestive of disseminated granulomatous disease.

Further imaging with CECT of the thorax and abdomen revealed multiple conglomerate necrotic mediastinal lymph nodes with calcifications; centrilobular nodules with a tree-in-bud appearance in the left lower lobe (Figure 1D); periportal and paraaortic lymphadenopathy; and multiple sub-centimetric non-enhancing hypodense lesions in the liver parenchyma consistent with hepatic granulomas (Figure 1C). The spleen was unremarkable. The hepatic and abdominal lymph node findings, together with bone marrow involvement, confirmed multisystem dissemination beyond the pulmonary compartment. These CT findings, particularly the tree-in-bud opacities and mediastinal lymphadenopathy, raised a strong suspicion for endobronchial spread of TB despite the chest X-ray showing only hilar lymphadenopathy without classic consolidation or cavitation. Chest X-ray is well recognised to have limited sensitivity for early, miliary, or paucibacillary TB, and CT of the thorax is substantially more sensitive for detecting early changes.

Based on the CT findings, bronchoalveolar lavage (BAL) was performed to obtain microbiological evidence of pulmonary TB. CBNAAT (Xpert MTB/RIF) was chosen given its high sensitivity in sputum-scarce patients. BAL CBNAAT returned positive for M. tuberculosis with no rifampicin resistance detected. First-line probe assay confirmed: rifampicin - sensitive; isoniazid (KatG) - sensitive; isoniazid (InhA) - sensitive.

A diagnosis of disseminated TB with presumptive tricuspid valve endocarditis was made. The patient was commenced on standard four-drug anti-tubercular therapy (ATT): isoniazid (5 mg/kg/day), rifampicin (10 mg/kg/day), pyrazinamide (25 mg/kg/day), and ethambutol (15 mg/kg/day) for a two-month intensive phase, followed by isoniazid and rifampicin for a 10-month continuation phase (total duration 12 months), in accordance with national and WHO guidelines for extrapulmonary TB. Adjunctive dexamethasone (8 mg twice daily, tapered over four weeks) was added, given the severity of systemic inflammation (hyperferritinemia, cytopenias, and hypoalbuminaemia), the presence of a large mobile vegetation with embolic risk, and by analogy with the documented benefit of corticosteroids in tuberculous pericarditis and meningitis (IMPI trial; Tuberculous Meningitis International Study Group [8]). We acknowledge that controlled trial data specifically supporting steroid use in tuberculous endocarditis are lacking; this represents an area of clinical inference rather than established evidence.

The patient responded rapidly and became afebrile within one week of commencing ATT. No further fever recurrence was reported. Surgical intervention was considered but deferred given the patient’s sustained clinical improvement and echocardiographic regression on medical therapy. The patient is currently completing the continuation phase of ATT with no complications.

## Discussion

Cardiac TB, encompassing myocardial, pericardial, and endocardial involvement, accounts for 0.14-2% of all TB cases. Among these, tuberculous valvular endocarditis is the rarest manifestation [9]. A review of the historical literature shows that tuberculous valvular endocarditis was reported only sporadically; by 1990, just 16 cases had been documented, and, in an analysis by Cope and colleagues, nearly all of those cases were diagnosed at autopsy [5]. It is usually seen in the context of disseminated TB, where hematogenous spread from a primary infection seeds multiple organs, including the heart valves. In most cases, myocardial involvement appears as a tuberculoma [10]. However, in our case, vegetations were identified on the tricuspid valve.

Before elaborating on the cardiac findings, it is important to distinguish the nature of TB in this patient. The infection was disseminated, not limited to the pulmonary compartment. M. tuberculosis was confirmed by BAL CBNAAT; bone marrow biopsy demonstrated epithelioid granulomas with Langhans-type giant cells, and CECT demonstrated hepatic granulomas and abdominal lymphadenopathy. This constitutes confirmed multisystem dissemination, a critical distinction that substantially strengthens the plausibility of hematogenous seeding of the tricuspid valve.

Regarding the cardiac diagnosis, we acknowledge that a positive BAL CBNAAT confirms M. tuberculosis infection but does not, in isolation, prove that the tricuspid valve vegetation is of tuberculous origin. We therefore classify this as presumptive tuberculous endocarditis, based on the totality of evidence: (1) microbiologically confirmed disseminated M. tuberculosis infection involving multiple organ systems; (2) a new tricuspid vegetation with no structural or degenerative explanation; (3) three pre-treatment blood culture sets negative for common bacterial and fungal pathogens (post-antibiotic cultures are diagnostically non-contributory and are not considered primary evidence); (4) absence of an alternative BCNE etiology despite broad workup; and (5) clinical improvement and echocardiographic regression of the vegetation following ATT alone, without additional anti-infective therapy directed at other organisms.

Applying the modified Duke criteria for IE [2], our case satisfies one major criterion (oscillating vegetation on echocardiography in the absence of an alternative anatomical explanation) and two minor criteria (fever >38°C; predisposing condition-endemic TB exposure with confirmed disseminated disease). This classifies the case as "Possible Infective Endocarditis" under the modified Duke schema - a designation that accurately reflects the absence of microbiological confirmation from valve tissue.

Several predisposing factors for tuberculous endocarditis have been noted in the literature, including immunosuppression, intravenous drug use, prosthetic valves, and congenital heart defects [11]. Our patient had none of these traditional risk factors. The patient had no prior documented immunosuppressive illness; however, a baseline leukopenia (WBC 2.85 × 10³/μL) with relative lymphopenia was present on admission. Although the leukopenia resolved following ATT, we cannot definitively establish whether it was a consequence of disseminated TB-associated bone marrow granulomatosis or a pre-existing immune vulnerability that predisposed the patient to severe disease, as no pre-illness complete blood count is available. This uncertainty reflects an important limitation and should caution against the uncritical use of the term "immunocompetent" in such cases.

The FNAC of the cervical lymph node showing only reactive lymphadenitis is an important diagnostic teaching point. FNAC has a reported sensitivity of only 40-60% for granulomatous lymphadenitis, owing to sampling limitations and the patchy distribution of granulomas [7]. A non-diagnostic FNAC must not be taken to exclude TB, particularly in a high-prevalence setting. In retrospect, an excisional biopsy, which carries substantially higher sensitivity for granulomatous pathology, would have been the preferred next diagnostic step following a non-diagnostic FNAC. This was deferred, given the patient’s clinical frailty; however, it represents a missed diagnostic opportunity that is acknowledged as a limitation.

The rationale for adjunctive dexamethasone warrants explicit discussion. The evidence base for corticosteroids in TB derives primarily from the IMPI trial (tuberculous pericarditis) [8], where corticosteroids reduced inflammatory pericardial complications, and from meningitis trials demonstrating reduced mortality and morbidity. In our patient, the combination of severe systemic inflammation (hyperferritinemia, cytopenias, hypoalbuminemia), a large mobile vegetation with embolic risk, and the clinicopathological analogy with pericardial TB disease informed this decision. We acknowledge that controlled trial evidence specifically supporting steroid use in tuberculous endocarditis is lacking, and the dose and duration used (8 mg twice daily, tapered over four weeks) were based on institutional experience and clinical judgment rather than an established evidence-based protocol. This should be noted as an area requiring further systematic study.

This case illustrates several important diagnostic and management lessons: the importance of CT over chest X-ray in guiding microbiological investigation for suspected TB; the low sensitivity of FNAC in granulomatous disease; the inferential nature of tuberculous endocarditis diagnosis without valve tissue; and the need to distinguish pulmonary from disseminated TB when framing diagnostic conclusions. The cases of Saboe et al. (2021) [12] and Abbara et al. [13] similarly illustrate the multisystem nature of TB cardiac involvement and the diagnostic challenges it poses and are consistent with the inferential diagnostic approach used in the majority of published cases.

Limitations

Several limitations of this report must be acknowledged. First, and most importantly, valve tissue was not obtained for histopathological or microbiological analysis; the diagnosis of tuberculous endocarditis is therefore presumptive, resting on confirmed multisystem disseminated TB, sterile pre-treatment blood cultures, echocardiographic vegetation, and clinical and echocardiographic response to ATT. Second, the cervical lymph node FNAC was non-diagnostic, showing only reactive lymphadenitis - a recognized limitation of FNAC in granulomatous disease. Excisional biopsy, which would have been more informative, was deferred due to clinical frailty. Third, formal serological exclusion of other BCNE pathogens, including Coxiella burnetii, Bartonella spp., Legionella, Mycoplasma, and Tropheryma whipplei, was not performed, and HACEK (Haemophilus, Aggregatibacter, Cardiobacterium, Eikenella, Kingella) extended cultures were not specifically documented; these represent incomplete exclusion of the full BCNE differential. Fourth, no pre-illness hematological data are available, precluding definitive determination of whether the baseline leukopenia predated or resulted from the disseminated TB. Fifth, long-term follow-up beyond six months is not available. These limitations are consistent with the inherent constraints of case report methodology and reflect the resource and clinical realities of a single-center experience.

## Conclusions

This case presents a rare and diagnostically challenging presentation of disseminated M. tuberculosis infection with concurrent tricuspid valve vegetation, most consistent with presumptive tuberculous endocarditis. In the absence of valve tissue confirmation, this diagnosis rests on the totality of clinical, microbiological, and imaging evidence: confirmed disseminated TB, sterile pre-treatment blood cultures, absence of an identified alternative BCNE aetiology, and echocardiographic and clinical response to anti-tubercular therapy alone. Clinicians in TB-endemic regions should include M. tuberculosis in the differential diagnosis of culture-negative endocarditis, particularly when systemic dissemination is demonstrated. A comprehensive microbiological workup, tissue diagnosis where clinically feasible, and application of the modified Duke Criteria should be standard practice in such presentations. A further prospective, multicenter study is required before generalised management recommendations, including the role of adjunctive corticosteroids, can be made for this rare condition.
